# Photoinactivation of Phage Phi6 as a SARS-CoV-2 Model in Wastewater: Evidence of Efficacy and Safety

**DOI:** 10.3390/microorganisms10030659

**Published:** 2022-03-19

**Authors:** Marta Gomes, Maria Bartolomeu, Cátia Vieira, Ana T. P. C. Gomes, Maria Amparo F. Faustino, Maria Graça P. M. S. Neves, Adelaide Almeida

**Affiliations:** 1 Department of Biology and CESAM, University of Aveiro, 3810-193 Aveiro, Portugal; mcarvalhogomes@ua.pt (M.G.); maria.bartolomeu@ua.pt (M.B.); catiavieira@ua.pt (C.V.); 2 Center for Interdisciplinary Investigation (CIIS), Faculty of Dental Medicine, Universidade Católica Portuguesa, 3504-505 Viseu, Portugal; 3 Department of Chemistry and LAQV-REQUIMTE, University of Aveiro, 3810-193 Aveiro, Portugal; gneves@ua.pt

**Keywords:** *Pseudomonas syringae*, photodynamic inactivation (PDI), environmental factors, porphyrins, wastewater, phage φ6, SARS-CoV-2, coronavirus, viruses

## Abstract

The last two years have been marked by the severe acute respiratory syndrome coronavirus 2 (SARS-CoV-2) pandemic. This virus is found in the intestinal tract; it reaches wastewater systems and, consequently, the natural receiving water bodies. As such, inefficiently treated wastewater (WW) can be a means of contamination. The currently used methods for the disinfection of WW can lead to the formation of toxic compounds and can be expensive or inefficient. As such, new and alternative approaches must be considered, namely, photodynamic inactivation (PDI). In this work, the bacteriophage φ6 (or, simply, phage φ6), which has been used as a suitable model for enveloped RNA viruses, such as coronaviruses (CoVs), was used as a model of SARS-CoV-2. Firstly, to understand the virus’s survival in the environment, phage φ6 was subjected to different laboratory-controlled environmental conditions (temperature, pH, salinity, and solar and UV-B irradiation), and its persistence over time was assessed. Second, to assess the efficiency of PDI towards the virus, assays were performed in both phosphate-buffered saline (PBS), a commonly used aqueous matrix, and a secondarily treated WW (a real WW matrix). Third, as WW is generally discharged into the marine environment after treatment, the safety of PDI-treated WW was assessed through the determination of the viability of native marine water microorganisms after their contact with the PDI-treated effluent. Overall, the results showed that, when used as a surrogate for SARS-CoV-2, phage φ6 remains viable in different environmental conditions for a considerable period. Moreover, PDI proved to be an efficient approach in the inactivation of the viruses, and the PDI-treated effluent showed no toxicity to native aquatic microorganisms under realistic dilution conditions, thus endorsing PDI as an efficient and safe tertiary WW disinfection method. Although all studies were performed with phage φ6, which is considered a suitable model of SARS-CoV-2, further studies using SARS-CoV-2 are necessary; nevertheless, the findings show the potential of PDI for controlling SARS-CoV-2 in WW.

## 1. Introduction

Severe acute respiratory syndrome coronavirus 2 (SARS-CoV-2) is the causative agent of the pandemic that was first discovered in December 2019 in Wuhan, China [1]. This new beta-coronavirus has an RNA genome ranging between 25 and 32 kb and a virion that is 118–136 nm in diameter [2].

Along with a respiratory infection, SARS-CoV-2 is also known to infect the gastrointestinal tract via the angiotensin-converting enzyme (ACE2) receptor that is expressed by epithelial cells in the gastrointestinal system [3]. The presence of SARS-CoV-2 has been observed in the feces of symptomatic and asymptomatic persons for long periods, even after negative detection through throat swabs and urine samples [4]. Although the possibility of SARS-CoV-2 being transmitted via the fecal–oral [5] or nasal–fecal pathways is still under discussion [3,6,7,8], this issue is a source of growing concern and can have negative environmental implications, namely, on water quality.

A large part of the wastewater (WW) released into the environment around the world is not adequately treated for the elimination of microorganisms and microbial entities, such as viruses [9]. In general, WW is only secondarily treated in wastewater treatment plants (WWTPs) and then released into rivers and seawater while still containing high concentrations of microorganisms and viruses [10]. These data highlight the potential risks of transmission of emerging microorganisms and microbial entities through discharge of WW into the environment, which implies the need for the development of efficient WW disinfection treatments [5,7]. Since SARS-CoV-2 is present in the intestinal tract, the virus is excreted in the feces of infected individuals, regardless of the severity of symptoms, and its RNA has been found in WWTPs [11]. The presence of SARS-CoV-2 RNA in sewage samples was already confirmed by studies from different countries, such as the Netherlands [12], China [13], EUA [14], Australia [15], Italy [16], among others [13,14,15,16]. Aside from the fact that the detection of this virus in WW has already proven to be a reliable indicator of the evolution of the pandemic over time, its presence cannot be ruled out as a potential risk factor for public health [17], since the incomplete removal of a variety of pathogenic microorganisms by WWTPs has led to known outbreaks across the world [18,19].

Tertiary disinfection treatments are already used; however, they can be expensive, toxic to aquatic organisms, and induce genetic damage to microorganisms and microbial entities [6,20]. As such, the development of new and safe technologies for WW disinfection must be taken into account.

Photodynamic inactivation (PDI) has already been shown to be an effective approach to inactivating Gram-positive and Gram-negative bacteria, viruses, fungi, and parasites using different types of photosensitizers (PSs), and it can be applied in clinical and non-clinical contexts [17,18,21,22]. This promising and selective therapy requires, aside from the PSs, the presence of an adequate light source and dioxygen (^3^O_2_) [19,23]. A key step in the photocatalytic cycle of PDI is an efficient conversion of the excited singlet state (of short duration) of the PS into a long-lived triplet state in order to allow the required generation of reactive oxygen species (ROSs) (e.g., ^1^O_2_, O_2_^•−^, OH^•^, H_2_O_2_), which lead to the irreversible oxidation of vital microbial constituents [24,25,26].

The purpose of this study was to evaluate if PDI can be considered an adequate approach for the inactivation of phage φ6 as a surrogate for SARS-CoV-2 in WW. PDI assays were performed under artificial white light and by using the tetracationic porphyrin 5,10,15,20-tetrakis(1-methylpyridinium-4-yl)porphyrin tetra-iodide [TetraPy(+)Me] as a PS. This positively charged PS was chosen due to its easy accessibility and recognized efficiency in inactivating different types of viruses [10,27]. 

In the PDI assays, the bacteriophage φ6 (or, simply, phage φ6) was used as a surrogate for SARS-CoV-2 after evaluating its survival rate under different laboratory-controlled environmental conditions. This phage is an enveloped RNA virus of the *Cystoviridae* family with spike proteins of a similar size (~80–100 nm) to those of SARS-CoV-2 and with an RNA genome of 13.5 kbp and a size of 75 nm [28]; it has previously been used as a suitable model for enveloped RNA viruses, such as coronaviruses (CoVs) [29]. The similar structures of both viruses, which are also associated with their similar survival in water and sewage and their behavior under particular environmental conditions, make phage φ6 a good, safe, and low-laboratory-cost SARS-CoV-2 surrogate [30,31,32,33]. Actually, some studies showed that the tolerance of phage φ6 to ultraviolet radiation inactivation [34], temperature, and humidity [35], as well as its recovery from hands [36] and persistence in water, sewage, and on surfaces [18,24,37], is similar to those observed for CoVs. In the context of the coronavirus disease (COVID-19) pandemic, some authors have used this bacteriophage as a model for SARS-CoV-2 to assess its persistence on porous and non-porous surfaces [30] and its survival in droplets dispersed on glass surfaces [38]. The same phage has also recently been used as a model for the coronavirus in surface disinfection studies mediated by ultraviolet light [39]. Furthermore, phage φ6 has also been suggested as a substitute for enveloped human viruses for the photodynamic inactivation approach under visible light [29]. In this study, we aimed to assess the persistence of phage φ6 over time in different environmental conditions (temperature, pH, salinity, and solar and UV-B irradiation) and the photodynamic efficacy of eradicating the virus in real WW matrices when compared with the efficacy in laboratorial conditions (phosphate-buffered saline solution—PBS). Finally, the safety of the whole PDI protocol was assessed by monitoring the viability of cultivable native marine water microorganisms after their contact with the PDI-treated effluent.

## 2. Materials and Methods

### 2.1. Experimental Design

As mentioned above, the PDI assays were carried out with phage φ6, an accepted surrogate model of SARS-CoV-2. This enveloped RNA virus is a member of the *Cystoviridae* family [40], with an RNA genome of 13.5 kbp and a size of 75 nm [28], and it multiplies in *Pseudomonas syringae* bacteria.

Before the photoinactivation tests, it was important to first understand the survivability of the virus in the environment. For this, phage φ6 was subjected to different laboratory-controlled environmental conditions (e.g., temperature, pH, salinity, and solar and UV-B irradiation) in order to assess its persistence over time. The values of temperature, pH, salinity, solar and UV-B irradiation were selected by taking into account the range of values for these variables in WW and in the environment during the year under temperate climatic conditions.

The potential of PDI to irradicate phage φ6 was first evaluated in vitro using an aqueous matrix of the commonly used phosphate-buffered saline (PBS) in order to select the best photoinactivation conditions to be used in WW disinfection. Then, the PDI assays were performed using real WW matrices, which were secondarily treated and collected on different days.

Once WW was discharged into the marine environment after treatment, the safety of PDI-treated WW was assessed through the determination of the viability of native marine water microorganisms after contact with the PDI-treated effluent. For the studies concerning the survivability of phage φ6 under different laboratory-controlled environmental conditions and for the PDI assays, at least three independent tests were done in different water samples that were collected on different days.

### 2.2. Bacterial Strain and Growth Conditions

As the bacterial host of phage φ6, we used *Pseudomonas sp.* [DSM 21482, purchased from Leibniz-Institute DSMZ—Deutsche Sammlung von Mikroorganismen und Zellkulturen GmbH (Braunschweig, Germany)]. The *Pseudomonas sp.* cells were cultivated under optimum growth conditions and controlled through the use of an orbital shaker (120 rpm) with the temperature set to 25 °C for 18 h in medium Tryptic Soy Broth (TSB; Liofilchem, Roseto degli Abruzzi, TE, Italy). After overnight growth, bacterial glycerol stocks were prepared in 10% glycerol and stored at −80 °C. Before each experiment, a bacterial stock was aseptically inoculated into 30 mL of fresh TSB. Then, the sample was incubated overnight as described above, until reaching a viable cell density of approximately 10^8^–10^9^ colony-forming units per milliliter (CFU mL^−1^).

### 2.3. Phage ϕ6 Preparation and Enrichment

Phage φ6 [DSM 21518, purchased from Leibniz-Institute DSMZ—Deutsche Sammlung von Mikroorganismen und Zellkulturen GmbH (Braunschweig, Germany)] suspensions were obtained from a previous phage stock prepared in SM buffer [0.1 M NaCl, 8 mM MgSO_4_, 20 mM Tris-HCl (reagents purchased from Sigma, St. Louis, MO, USA), 2% (*w*/*v*) gelatin, pH 7.5] using the bacterium *Pseudomonas* sp. as a host. A 2 mL aliquot of phage stock and 1 mL of the overnight grown bacteria suspension of *Pseudomonas* sp. were added to 50 mL of SM buffer. The obtained suspension was incubated overnight under orbital shaking (60 rpm) at a controlled temperature of 25 °C. The preparation was centrifuged at 12,000× *g* rpm for 10 min, and the obtained supernatant was filtered through a membrane with a pore size of 0.22 µm (Merck Millipore, Darmstadt, Germany) in order to remove the remaining bacterial debris or intact bacteria. 

The phage titer was determined by the double-layer agar method and the phage suspension was maintained at 4 °C. For the phage titration, successive dilutions of the phage suspension were made in phosphate-buffered saline [PBS; 137 mM NaCl, 2.7 mM KCl, 8.1 mM Na_2_HPO_4_·2H_2_O, 1.76 mM KH_2_PO_4_ (reagents purchased from Sigma, St. Louis MO, USA), pH 7.4], and to 5 mL of the TSB 0.6% top agar layer were added 500 µL of the phage and 200 µL of *Pseudomonas* sp. suspensions, which were placed on a Petri plate with TSA. The plates were incubated for 18 h at 25 °C and formed plaques, which were expressed as plaque-forming units per milliliter (PFU mL^−1^); the stock solution of the phage was calculated to be 10^8^–10^9^ PFU mL^−1^. A spot test was also performed to confirm the phage stock’s purity. For that, 5.0 mL of the TSB 0.6% top agar layer with 200 µL of bacteria was added to a plate with Tryptic Soy Agar (TSA; Liofilchem, Roseto degli Abruzzi, TE, Italy), and after, 20 µL of the phage stock was added. The plate was incubated as described above.

### 2.4. Wastewater Sample Collection

Composite wastewater samples were collected from a wastewater treatment plant (WWTP) located at the littoral center of Portugal. The WWTP receives wastewater from both domestic and industrial facilities. The WW composite collection was the result of a series of individual samples taken from the secondary treatment station over a total period of 24 h. The composite samples were collected on different days in autumn 2020. After the collection, the WW samples were kept in the dark and refrigerated at 4 °C until further use, within a maximum period of one week.

### 2.5. Phage φ6 Survival Assessment under Different Environmental Conditions

The effects of temperature, pH, salinity, and radiation (UV-B and sunlight) on the viability of phage φ6 (initial phage concentration of 10^7^ PFU mL^−1^) were tested in 10 mL of WW filtered by 0.22 µm pore membranes [mixed cellulose ester (MCE) membrane; Millipore, Bedford, MA, USA] and sterilized (with an autoclave procedure). During the experiments, aliquots of the samples were collected to determine the phage titer. The aliquots were serially diluted in PBS and plated with the double-layer agar method. Plates were incubated at 25 °C for 18 h.

At least three independent trials were performed for each condition. The end of the experiments was considered when non-detection of viral lysis plaques was achieved.

#### 2.5.1. Temperature Experiments

To evaluate the effect of temperature on phage viability, the phage suspension was added to the previously prepared WW samples, and the samples were maintained at defined and constant temperatures of 17, 25, and 37 °C in an incubating chamber and protected from light. To assess the effects of the selected temperatures, aliquots were collected every day during the first week (days 0, 1, 2, 3, 4, 5, 6, and 7 of WW incubation), followed by a once-a-week collection until the end of the experiments.

#### 2.5.2. pH Experiments

In order to evaluate the effect of pH on phage viability, suspensions of phage φ6 were added to the previously prepared WW samples with adjusted pH values of 6.0, 8.0, and 9.0. To obtain the desired pH values, acidic/basic solutions (HCl/NaOH) were added to the WW samples as needed. During these experiments, the temperature of the samples was kept at 17 °C. To assess the effect of the selected pH, aliquots were collected every day during the first week (days 0, 1, 2, 3, 4, 5, 6, and 7 of incubation), followed by a once-a-week collection until the end of the experiments.

#### 2.5.3. Salinity Experiments

In order to evaluate the effect of salinity on phage viability, phage suspensions were added to the previously prepared WW samples with salinity values adjusted to 34 and 15 g kg^−1^ by adding Tropic Marin^®^ Pro-Reef (Tropic Marin^®^, Wartenberg, Germany) as an artificial seawater medium. The samples were maintained at 17 °C during the experiments. A control sample was made in fresh WW without changing the salinity value and was kept at the same conditions. To assess the effect of the selected salinity, aliquots were collected every day during the first week (days 0, 1, 2, 3, 4, 5, 6, and 7 of incubation), followed by a once-a-week collection until the end of the experiments.

#### 2.5.4. UV-B Irradiation Experiments

To evaluate the effect of UV-B irradiation (280–320 nm) on phage viability, a UVB Broadband 20 W/12 RS TL lamp (Philips, Amsterdam, the Netherlands) was used and placed at a distance of 25 cm from the samples. The experiments were performed in the previously prepared WW samples and PBS, and the temperature was controlled during the experiment and maintained at 17 °C. Control samples were incubated in the same conditions as the test samples, but were not exposed to UV-B radiation. To assess the effect of UV-B irradiation, aliquots were collected after 0, 2, 4, 6, 8, 10, and 12 h of light exposure.

#### 2.5.5. Solar Radiation Experiments

The effect of solar radiation on phage viability was evaluated by exposing the phage φ6 suspensions added to the WW samples to natural solar radiation. Control samples were assessed in parallel with the test samples under the same conditions, but were not exposed to solar radiation. During these experiments, the solar irradiance ranged between 46.2 and 91.1 mW cm^−2^ on spring days with ambient temperatures ranging between 13.5 and 16.7 °C; these solar irradiances and temperatures were monitored through the meteorology website CliM@UA of the University of Aveiro, where the tests were carried out. Aliquots were collected after established periods of natural solar irradiation (0, 2, 4, and 6 h).

### 2.6. Photodynamic Inactivation (PDI) Treatments

The PS 5,10,15,20-tetrakis(1-methylpyridinium-4-yl)porphyrin tetra-iodide [TetraPy(+)Me] was prepared according to the literature [41]. The purity was confirmed through thin-layer chromatography and ^1^H NMR. The stock solution in DMSO (500 µM) was protected from light and stored at room temperature.

The assays were performed in PBS and in 0.22 µm filtered WW in 6-well plates with a final volume of suspension of 5.0 mL per sample. For the PBS or the filtered WW, a determined phage stock volume was added to each sample well to achieve the initial phage concentration of ca. 10^7^–10^8^ PFU mL^−1^. TetraPy(+)Me was added to reach a final concentration of 5.0 µM. Light and dark controls were performed alongside the PDI samples; in the light controls (LCs), a phage suspension in PBS or filtered WW was exposed to light without TetraPy(+)Me addition; in the dark controls (DCs), the phage suspension in PBS or filtered WW, which contained TetraPy(+)Me at the same concentration as in the samples, was protected from light by being wrapped in aluminum foil during the light exposition. Before the irradiation, samples and controls were subjected to a pre-irradiation period of 10 min in the dark with shaking at room temperature to promote the binding of the PS to the phage φ6. Then, the samples and LCs were exposed to a white light-emitting diode (LED) system (EL^®^MARK, 20 W, ~230 V, 1400 lm, 5500 K, and ~50 Hz) at an irradiance of 50 mW cm^−2^, which was measured and adjusted with a power meter [FieldMaxII-Top (Coherent, Santa Clara, CA, USA)] connected to a high-sensitivity PS19Q sensor (Coherent, Santa Clara, CA, USA). During the experiments, the samples were magnetically stirred, and aliquots of 100 µL of samples and controls were taken at 0, 5, 10, 15, and 30 min, serially diluted in PBS, and plated on Petri dishes with the drop-plating method. The Petri dishes were previously prepared with TSA and a layer of TSB 0.6% top agar layer with the phage host *Pseudomonas* sp. for the monitoring of phage survival. At least three independent assays were performed for each condition.

### 2.7. Effect of the PDI-Treated Effluent on Native Marine Water Microorganisms

Samples (ca. 5 L) of coastal marine water were collected in the littoral center of Portugal in spring 2021. The samples were filtered with a membrane with a pore size of 1.2 µm to remove the suspended matter, followed by a second filtration with a membrane with a pore size of 0.22 µm [mixed cellulose ester (MCE) membrane; Millipore, Bedford, MA, USA] to remove the remaining native bacteria, mold, and yeast. The marine water sample that was filtered two times was sterilized with moist heat to ensure the inactivation of residual microorganisms and other biological entities (such as viruses) whose dimensions did not allow their retention by the membranes used. The sterilized marine water samples were stored and protected from light at 4 °C until further use within a maximum period of one week.

On the day of the assay, new coastal marine water samples were collected (2.0 L). The number of total cultivable microorganisms was determined using Plate Count Agar (PCA, Liofilchem, Roseto degli Abruzzi, TE, Italy) as a culture medium; a volume of 1.0 mL of the collected marine water was plated through incorporation into the PCA medium on Petri dishes; the plates were incubated for 18 h at 25 °C; after incubation, the contents of the plates were counted and the results were expressed in CFU mL^−1^. The collected marine water samples were pre-filtered with a 1.2 µm membrane [mixed cellulose ester (MCE) membrane; Millipore, Bedford, MA, USA] to remove the suspended matter. After the pre-filtration step, a volume of 500 mL of the pre-filtered samples was filtered with a membrane with a pore size of 0.22 µm, and the retained content of the filter was resuspended in 5.0 mL of the sterilized marine water that was previously prepared in order to concentrate the native marine water microorganisms, hereinafter referred to as the “native marine microorganism concentrate” for ease of identification. The total number of cultivable microorganisms was quantified again, as previously described, in the PCA medium, and the prepared suspension was stored until further use within a maximum period of 24 h.

PDI experiments were carried out under the same conditions as those mentioned in Section 2.6 [the PS TetraPy(+)Me was used at a concentration of 5.0 µM under white-light irradiation at an irradiance of 50 mW cm^−2^ for 30 min], but without the addition of any biological entities. To the previously prepared suspension of resuspended native microorganisms that were added to the sterile marine water, a determined volume of PDI-treated WW was added, and the following samples were performed:

(i). non-irradiated control of the native marine microorganism concentrate (DC marine water);
(ii). irradiated (50 mW cm^−2^) control of the native marine microorganism concentrate (LC marine water);
(iii). non-irradiated controls of filtered WW added to the native marine microorganism concentrate in the ratios of 1:2, 1:10, 1:100, and 1:1000 (WW: native marine microorganism concentrate), DC-diluted;
(iv). Irradiated (50 mW cm^−2^) controls of filtered WW added to the native marine microorganism concentrate in the ratios of 1:2, 1:10, 1:100, and 1:1000 (WW: native marine microorganism concentrate), LC-diluted;
(v). non-irradiated samples with PDI-treated filtered WW added to native marine microorganism concentrate in the ratios of 1:2, 1:10, 1:100, and 1:1000 (WW: native marine microorganism concentrate), S (dark);
(vi). irradiated samples (50 mW cm^−2^) with PDI-treated filtered WW added to native marine microorganism concentrate in the ratios of 1:2, 1:10, 1:100, and 1:1000 (WW: native marine microorganism concentrate), S (light).

The replicates were made for a total volume of 10 mL, and the assays were carried out at a constant temperature of 17 °C. The irradiation period of the samples and controls lasted 24 h, and aliquots of the samples and controls were collected at 0, 6, and 24 h.

From each treated and control sample, tenfold serial dilutions were prepared in sterile PBS (10^0^ to 10^−6^). Aliquots of 100 µL were pour-plated in PCA. The plates were incubated at 25 °C for 18 h, and the number of colony-forming units was counted. At least three independent assays were performed.

### 2.8. Statistical Analyses

A statistical analysis was performed using the GraphPad Prism software program. The Kolmogorov–Smirnov test was used to check the normal distribution of the data, and homogeneity of variances was tested with the Brown–Forsythe test. ANOVA and Tukey’s multiple comparisons test were applied to assess the significance of the differences among the tested conditions. Differences corresponding to *p* < 0.05 were considered significant. Three independent assays were performed for each condition.

## 3. Results

### 3.1. Assessment of the Effect of Environmental Factors on Phage φ6 Viability

#### 3.1.1. Temperature Experiments

**Figure 1:**
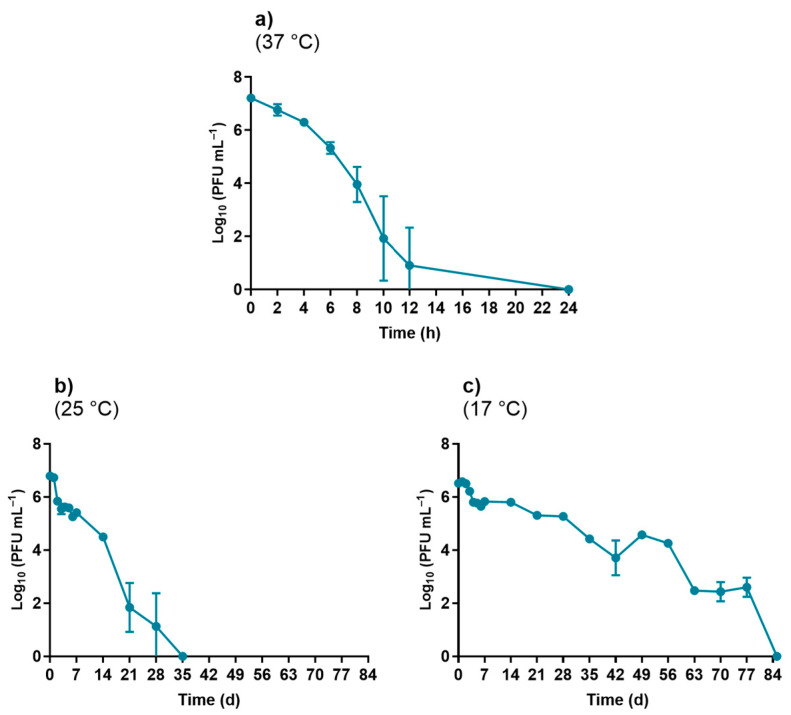
Survival of phage φ6 following exposure to different temperature values: (**a**) 37 °C (time units in hours), (**b**) 25 °C (time units in days), and (**c**) 17 °C (time units in days). Data points represent the average of three independent experiments; error bars represent the standard deviation. Lines just connect the experimental points.

The results summarized in Figure 1 show that the viability of phage φ6 is temperature dependent. The highest rate of decrease in viability was observed in the assays performed at 37 °C, followed by the ones at 25 °C and then at 17 °C. After 24 h at 37 °C, the viability of the phage φ6 decreased to the detection limit of the method, although a sharp decrease of 6.0 log_10_ PFU mL^−1^ was detected after 12 h (Figure 1a). In the assays performed at lower temperatures, the decrease in viability to the detection limit of the method was much slower, and it required 35 days for the assays performed at 25 °C (Figure 1b) and 84 days for the ones performed at 17 °C (Figure 1c).

Considering these variations, the subsequent assays were performed at 17 °C (vide infra the justification for the selection of this temperature).

#### 3.1.2. pH Experiments

**Figure 2:**
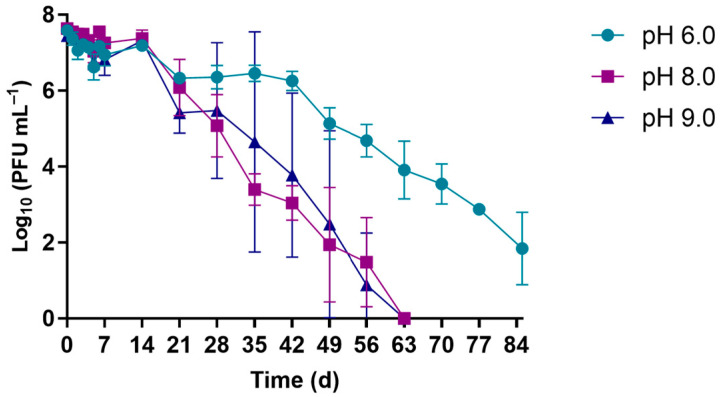
Survival of phage φ6 at 17 °C and following exposure to different pH values (6.0, 8.0, and 9.0). Data points represent the average of three independent experiments; error bars represent the standard deviation. Lines just connect the experimental points.

The effects of different pH values (6.0, 8.0, and 9.0) on the viability of phage φ6 with the samples maintained at 17 °C are summarized in Figure 2. The results show that the selected phage behaved similarly in solutions at pH 8.0 and 9.0, where a decrease of 7.5 log_10_ PFU mL^−1^ was observed after 63 days (9 weeks) (Figure 2) (ANOVA, *p* > 0.05). At pH 6.0, a decrease of 5.7 log_10_ PFU mL^−1^ in the phage viability was only observed after 84 days (3 months) (Figure 2).

#### 3.1.3. Salinity Experiments

**Figure 3:**
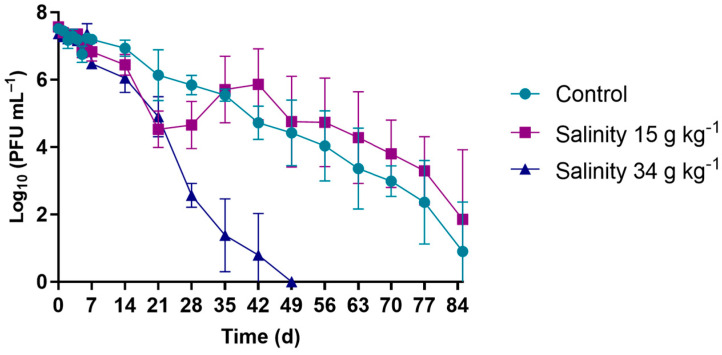
Survival of phage φ6 at 17 °C following exposure to different salinity values. Data points represent the average of three independent experiments; error bars represent the standard deviation; phage controls had no change in salinity content. Lines just connect the experimental points.

In the viability assays, where the survival of phage φ6 was studied under different salinity conditions (Figure 3), a decrease in the phage viability of 7.3 log_10_ PFU mL^−1^ was observed after 49 days (7 weeks) for the salinity of 34 g kg^−1^. For the control (WW samples without salt addition) and for the solution adjusted to a salinity of 15 g kg^−1^, the decreases in phage viability were 6.4 and 5.7 log_10_ PFU mL^−1^, respectively, only after 84 days (3 months) (ANOVA, *p* > 0.05).

#### 3.1.4. UV-B Exposure Experiments

**Figure 4:**
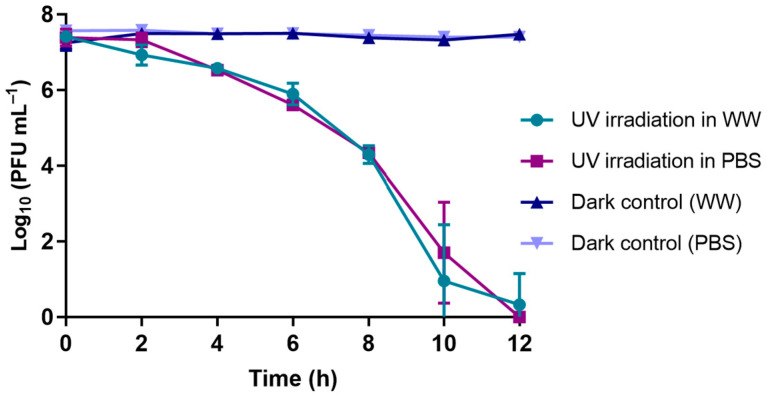
Survival of phage φ6 at 17 °C following exposure to UV-B radiation (290–320 nm). Assays were performed in PBS and WW. Data points represent the average of three independent experiments; error bars represent the standard deviation; in dark controls, the phage suspensions were not exposed to UV-B radiation. Lines just connect the experimental points.

The results of the survival of phage φ6 when exposed to UV-B irradiation (290–320 nm) show that the assays performed in either WW or PBS showed similar decreases in viability after 12 h of exposure (ANOVA, *p* > 0.05), with viability reductions of 7.2 log_10_ PFU mL^−1^ and of 7.5 log_10_ PFU mL^−1^ (Figure 4).

In the same experiment (Figure 4), it was found that the concentration of phage φ6 when not exposed to UV-B radiation remained constant for the same period in both the PBS and WW matrices (ANOVA, *p* > 0.05).

#### 3.1.5. Solar Radiation Experiments

**Figure 5:**
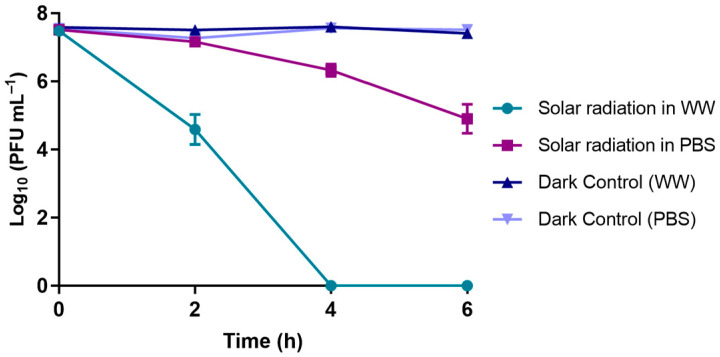
Survival of phage φ6 following exposure to a solar irradiance from 46.2 to 91.1 mW cm^−2^ on a day with an ambient temperature ranging from 13.5 to 16.7 °C. Assays were performed in PBS and WW. Data points represent the average of three independent experiments; error bars represent the standard deviation; phage controls were not exposed to solar radiation. Lines just connect the experimental points.

The results obtained when phage φ6 was exposed to solar radiation (at an irradiance from 46.2 to 91.1 mW cm^−2^, with an ambient temperature ranging from 13.5 to 16.7 °C) in either PBS or WW are summarized in Figure 5. In PBS, the abundance of the phage decreased by 2.6 log_10_ PFU mL^−1^ after 6 h of irradiation when compared to the phage control. In WW, the abundance reduction was higher; a decrease of 7.5 log_10_ PFU mL^−1^ was observed when compared to the respective control after 4 h of exposure to solar radiation (ANOVA, *p* < 0.05).

### 3.2. Evaluation of the Viability of Phage φ6 after PDI in the Presence of TetraPy(+)Me

**Figure 6:**
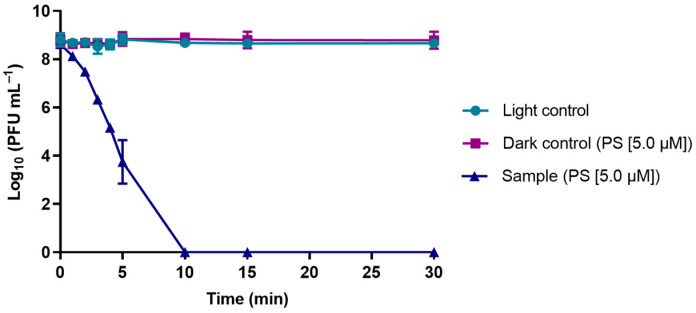
Survival of bacteriophage φ6 at 17 °C in PBS during PDI with TetraPy(+)Me at 5.0 µM after irradiation with a light-emitting diode (LED) at an irradiance of 50 mW cm^−2^ for 30 min. Data points represent the average of three independent experiments; error bars represent the standard deviation; phage controls were not exposed to solar radiation. Lines just connect the experimental points.

**Figure 7:**
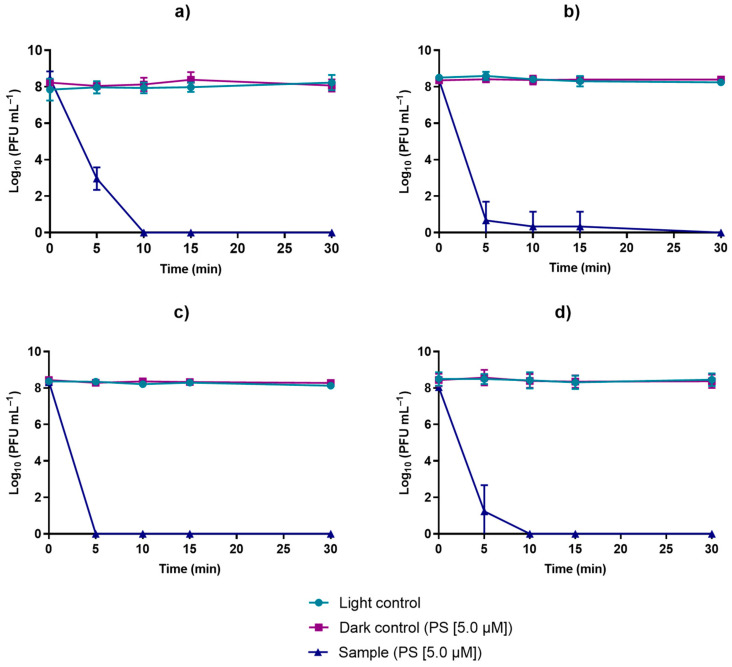
PDI using the tetracationic porphyrin TetraPy(+)Me and with light (LED) at 50 mW cm^−2^, in the inactivation of bacteriophage φ6 in WW collected on three different days: (**a**) October, (**b**) 16 December, and (**c**) 18 December. In (**d**), the mean of the results presented in (**a**–**c**) is presented. Data points represent the average of three independent experiments; error bars represent the standard deviation; phage controls were not exposed to solar radiation.

The results obtained from the PDI assays mediated by TetraPy(+)Me that were performed in PBS and in WW are summarized in Figure 6 and Figure 7, respectively. In all of these assays, the TetraPy(+)Me concentration was 5.0 µM and the irradiation was performed using a white light-emitting diode (LED) at an irradiance of 50 mW cm^−2^ at 17 °C. The PDI assays in PBS were performed at a pH of 7.4, and the PDI assays in WW were performed in a pH range of 6.0–8.0.

The data obtained from the assays performed in PBS show that phage φ6 was efficiently inactivated by PDI after 5 min of treatment (more than 3.0 log), reaching the detection limit of the method after 10 min of PDI treatment (reduction of >8.0 log) when compared with the controls for the same time (ANOVA, *p* < 0.05) (Figure 6).

In the PDI assays carried out in WW under the same conditions as with the PBS treatments, WW collected on three different days was used (October, 16 December, and 18 December, 2020). The phage φ6 was efficiently inactivated (reduction of >8.0 log) by PDI after 30 min of treatment in all assays (Figure 7). In the studies carried out in the WW collected in October, the phage was inactivated after 10 min of treatment (Figure 7a) to the detection limit of the method. In the case of the water collected on December 16, the photoinactivation to the detection limit of the method occurred after 30 min of irradiation; however, at 5 min, there was already a phage decrease of 7.7 log_10_ PFU mL^−1^ (Figure 7b). In the case of the WW collected on December 18, the photoinactivation to the detection limit of the method was observed after 5 min of treatment (Figure 7c). However, these small apparent differences were not relevant when analyzing the pooled data of the three water samples (Figure 7d). Figure 7d shows that, at the end of 10 min, there was an average decrease of >8.0 log. Analyzing the differences between the results of the three samples (Figure 7a–c), it can be seen that, after 10 min of treatment, there were no significant differences in the inactivation efficiency (ANOVA, *p* < 0.05), which reinforced the efficiency of the aPDT in the inactivation of viruses in WW.

### 3.3. Effect of the PDI-Treated Effluent on Cultivable Native Marine Water Microorganisms

The survival of cultivable native marine water bacteria, mold, and yeast in the presence of PDI-treated filtered WW was evaluated using different ratios of WW to native marine microorganism concentrate (1:2, 1:10, 1:100, and 1:1000). These assays, referred as samples (S), were irradiated with the white light source at an irradiance of 50 mW cm^−2^, and the survival of the native marine microorganisms was compared with that in the adequate dark and light controls (see the details in Section 2.7 of the experimental part).

**Figure 8:**
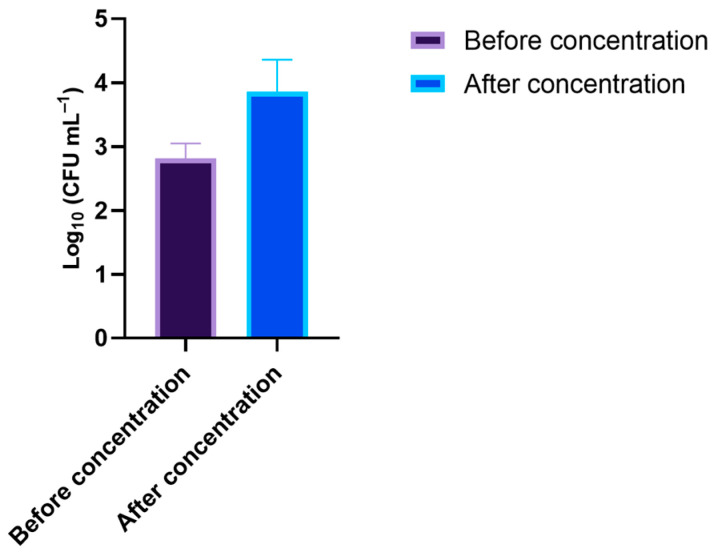
Quantification of the total cultivable native marine microorganisms before and after the concentration process.

On the day of the assay, new coastal marine water samples were collected, the total cultivable native marine microorganisms were quantified, and the value of 2.8 log_10_ CFU mL^−1^ was obtained. After the concentration process, the total cultivable native marine microorganisms were quantified again, revealing the increase in the total cultivable microorganisms to 3.9 log_10_ CFU mL^−1^ (Figure 8).

**Figure 9:**
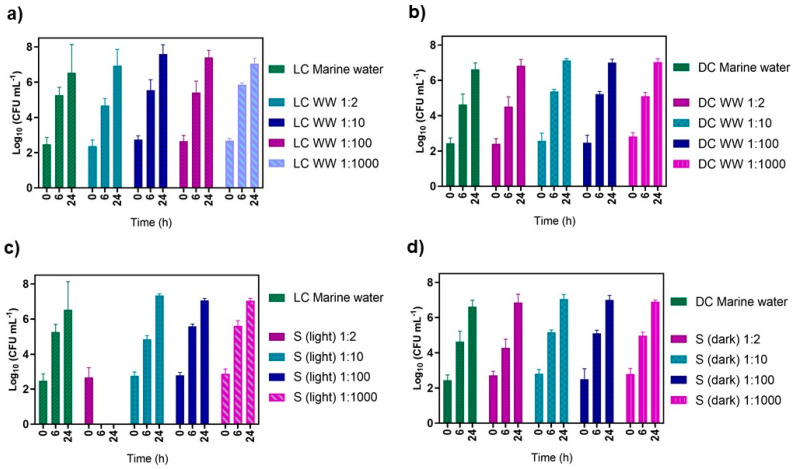
Survival of cultivable native marine water microorganisms after exposure to light in WW treated previously with 5.0 μM of TetraPy(+)Me and white light (50 mW cm^−2^): (**a**) light controls: LC marine water, LC WW (at different ratios); (**b**) dark controls: DC marine water, DC WW (at different ratios); (**c**) samples exposed to light: S (light) (at different ratios); (**d**) samples kept in the dark: S (dark) (at different ratios). Data values represent the average of three independent experiments.

The results summarized in Figure 9 show that only the light assays in which a ratio of PDI-treated filtered WW to native marine microorganism concentrate of 1:2 ratio was used affected the survival of native marine water microorganisms (Figure 9c) (ANOVA, *p* < 0.05). The bacterial concentrations of the other samples at different ratios were constant throughout the 24 h of the experiment (ANOVA, *p* > 0.05). The survival of native marine water microorganisms in the other assays involving all dark controls and non-PDI-treated filtered WW:native marine microorganism concentrate were also not affected (Figure 9a,b,d) (ANOVA, *p* < 0.05).

## 4. Discussion

Since the main objective of our work was to evaluate the potential of PDI to inactivate phage φ6 (recognized as a predictive model of the mammalian virus SARS-CoV-2) in wastewater (WW), it was crucial to first know the viability of the virus in different environmental conditions, namely, temperature, pH, salinity, and light conditions.

Several studies showed that temperature can affect the viability of viruses [26,27]. This fact prompted us to evaluate the viability of phage φ6 in WW at different temperatures (37, 25, and 17 °C). In this selection, we took into account that 17 °C is the closest temperature to the annual average temperature of seawater in central coastal Portugal [28,29,34], where WW is released after treatment, as well as in wastewater treatment plants (WWTPs). The data obtained in this study and summarized in Figure 1 corroborate the data previously obtained in PBS by Pinheiro et al. [42], showing that the virus is less viable at higher temperatures (37 °C) than at lower temperatures (17 °C); at 17 °C, the phage remains active for up to 3 months (84 days) (ANOVA, *p* < 0.05). Since 17 °C is closest to the temperature of WWTPs and of the seawater where the wastewater will be released, the obtained results confirm the need for an effective way of inactivating the viruses in WW. The phage φ6 is an enveloped RNA virus that has often been studied as a surrogate for coronaviruses due to its similar survival in water, sewage, and on surfaces, as well as its behavior when exposed to established environmental conditions (temperature) [30,31,32,33,35]. Our results showed a longer survival of phage φ6 at lower temperatures (17 and 25 °C) and the opposite at the highest tested temperature (37 °C). Although 37 °C is an unusual temperature for some countries, for others, it is not, and it was important to see the impact of this extreme temperature on the survival of phage φ6. As previously reported for phage φ6 and other viruses, the decrease in viral viability associated with increasing temperature could be due to an increased activity of extracellular enzymes [33,43,44].

pH is another important factor that has been recognized to influence phage survival in the environment [45]. The pH values tested in this study (6.0, 8.0, and 9.0) represent the pH range that is usually allowed for treated effluents that are discharged into the environment (6.5–8.5) [46], as well as the global average pH at the ocean’s surface (around 8.1) [47]. The data obtained (Figure 2) showed that the phage remained viable in WW over several weeks for all of the pH values: 7 weeks for pH 8.0 and 9.0 and at least 12 weeks for pH 6 (ANOVA, *p* < 0.05). These results reiterate the need for viral inactivation in WW before its discharge into the environment. 

Considering that marine water is one of the places where WW is released after treatment, salinity is also an important factor to take into account. As such, the phage was incubated in WW with salinity values of 15 and 34 g kg^−1^, corresponding, respectively, to the values of brackish and marine water—in these samples, the pH of the WW samples was not altered, naturally varying in the range of 6.0–8.0, and the test samples were maintained at 17 °C during the experiments. Both brackish and marine water salinities were considered to take the location of an underwater sewage outlet located at the coastal center of Portugal into account (40°40′59.131′’ N 8°46′47.249′’ W)—this underwater sewage outlet releases the treated wastewater into the seawater environment, but near a lagoon system, Ria of Aveiro, with brackish water. In these assays, it was found that the phage viability decreased in more saline environments (ANOVA, *p* < 0.05), remaining, however, viable for 7 weeks (Figure 3).

In the environment, solar irradiation—more specifically, UV radiation—is recognized as the most important factor of viral infectivity loss [33,38]. Solar radiation can directly affect viruses by degrading proteins, altering their structure, and damaging nucleic acid, leading to decreased infectivity [48]. Among the types of solar radiation, UV-B (wavelengths between 280 to 320 nm) is the most significant in the inactivation of microorganisms, considering that UV-A (wavelengths, 320 to 400 nm) has a lower antimicrobial effect, and UV-C, although used for its germicidal effects, is absorbed in the early stratosphere and does not reach the Earth’s surface [49,50,51,52]. Therefore, in this study, UV-B was selected to evaluate the effect of UV radiation on viruses in the environment.

Effectively, as in the study of Pinheiro et al. [42], where a decrease in the abundance of phage φ6 particles in PBS was observed when exposed to UV-B radiation (ca. 7 log_10_ PFU mL^−1^ after 8 h), in our study, a decrease of >8.0 log_10_ PFU mL^−1^ was also found until reaching 12 h of radiation (Figure 4). However, the phage particles under solar radiation remained viable in PBS (with a small decrease in phage viability of 2.6 log_10_ PFU mL^−1^ after 6 h of irradiation); in WW, the viability of the phage particles had a greater decrease of 7.6 log_10_ PFU mL^−1^ in a treatment with a shorter time of 4 h (Figure 5). These differences in phage viability under solar radiation when in different water matrices (ANOVA, *p* < 0.05) show that certain compounds in WW samples may act as photosensitizers (e.g., dissolved organic matter, antibiotics, nitrates) [53], making them responsible for the higher phage inactivation when compared with that in the viability tests performed in PBS. The dissolved matter can be photoactivated under solar irradiation, resulting in the production of reactive oxygen species (e.g., ^1^O_2_, OH^•^), which could affect the survival of the phage [54].

Since phage φ6 is composed of RNA, proteins, and phospholipids [55], and these molecules are potential targets for viral photoinactivation [46,47], it is expected that the virus is sensitive to light, even though there is no direct evidence of the existence of endogenous PSs [43,44]. According to the literature, phage φ6 has already proved to be sensitive to blue irradiation [56,57] without any added PSs. Although the phage does not contain endogenous PSs, it is speculated that the virus may carry bacterial sensitizing dyes from its host (*P. syringae*) when it assembles its envelope [57].

The physical and chemical environmental conditions evaluated in this study (temperature, pH, salinity, and solar and UV-B irradiation) are factors that have been continuously reported to highly influence the viability and transmission of viruses in wastewater (WW) [32,45]. The viral persistence in WW is also thought to be influenced to some extent by other environmental conditions, such as the presence of solvents, detergents, or other chemicals and other particulate and dissolved organic matter in this matrix [32,33,58]. Considering these conditions, and to represent their natural variations, in this study, the WW samples used in the phage φ6 survival assessment were collected on different days. Nevertheless, for each tested condition, the bacteriophage presented similar survival among the different water samples, indicating that it was not significantly impacted by the non-measured factors and showing that the variation of the chosen parameters (temperature, pH, salinity, and solar and UV-B irradiation) had a higher influence on the bacteriophage’s survival than other inhibitors that were possibly present (and not quantified) in the water samples. The WW samples were processed in some way during their preparation process for the viability assays (filtration and autoclaving), as well as for the PDI assays (filtration), in order to remove the microorganisms in the collected raw WW samples. These processes can alter the microbiota and the particulate organic matter content of the WW and, consequently, affect the survival of the phage in WW; however, the dissolved matter in WW, which was estimated to have the highest impact on the viruses’ survival, was not removed by the filtration process [59]. In addition, although some studies hypothesized that microorganisms may influence viruses’ viability through microbial predation and production of bacterial proteases [60,61], other studies demonstrated similar viral decay rates between pasteurized and non-pasteurized WW, suggesting that the viral viability may not always be influenced by biological factors of WW [33] in secondarily treated WW [processed by filtration (Figure 7)].

PDI has been the subject of many in vitro and ex vivo studies, and it was already used in the inactivation of other viruses, such as in the treatment of Herpes Simplex Viruses (HSVs) [50,62], Human Papilloma Virus (HPV) [48,63,64,65], and Varicella–Zoster Viruses (VZVs) [66]. The efficiency of PDI for the treatment of bacterial lung infections has also been studied [67,68]. Our research group previously reported the efficiency of this approach in inactivating pathogenic bacteria and mammalian model viruses in WW [10,69,70]. However, the application of this technique to the elimination of SARS-CoV-2 and its surrogates has yet to be studied. It was also shown by Tomb et al. [42,45] that phage φ6 is inactivated by PDI using exogenous PSs, namely porphyrins, during illumination with visible light. This effect is explained by the production of ROSs (formed during the irradiation process), which oxidize the envelope lipids, the proteins of the capsid, and the nucleic acids of the viruses [71].

Regarding WW, although, to the best of our knowledge, there are no experimental studies in the literature on the ability of PDI to inactivate SARS-CoV-2 in WW, it is expected that PDI can be an efficient approach to disinfecting WW. In addition to the fact that PDI has already demonstrated its ability to inactivate other viruses, namely, enveloped viruses, such as CoVs [71,72], different PSs have already been shown to be effective in the photodynamic inactivation of viruses in secondarily treated WW [14,54].The results obtained throughout this work support the theory that PDI can be an efficient alternative for the inactivation of SARS-CoV-2 in secondarily treated WW as a tertiary treatment approach to WW disinfection, as very promising results were obtained: A viral load (phage φ6 used as a SARS-CoV-2 surrogate) > 8 log_10_ PFU mL^−1^ was photoinactivated after just 10 min of treatment using the porphyrin PS TetraPy(+)Me in the micromolar range (5.0 µM) in secondarily treated WW (processed by filtration) (Figure 7). After collection, the secondarily treated WW samples were processed by filtration in order to remove the microorganisms present in the raw WW samples. As said before, the dissolved organic matter remained in the processed WW samples. Chemical factors, such as the presence of disinfectants, detergents, photosensitizer molecules, and other compounds in WW, may influence the PDI of viruses [43,69].

We hypothesized that physical and chemical factors could greatly affect the survival of viruses in the environment and, consequently, may aid or impair the efficiency of PDI [32,45,73]. Considering the presence of chemical compounds in WW and their consequent influence on viruses’ viability, we evaluated the survival of phage φ6 in PBS and WW matrices under solar radiation and in the dark. No variation in phage viability was observed in the dark, indicating that non-photosensitive compounds did not affect the survival of the phage in the evaluated time (6 h). On the other hand, phage viability was reduced in both matrices under solar radiation, with the highest decrease observed for WW. These results indicate that certain compounds in WW samples may act as photosensitizers, which are responsible for the higher phage inactivation when compared with the viability experiments performed in PBS. Furthermore, to understand whether the natural variations in WW composition could affect PDI efficiency, the WW samples used in PDI experiments were collected on different days. No significant differences were observed between the PDI of phage φ6 in the three independent assays, indicating that the natural variation in the contents of chemical compounds and other parameters in this matrix did not significantly impact the efficiency of PDI under the tested conditions (irradiance power, PS concentration, and consequent time of irradiation exposure).

The tertiary treatments that are mainly used to disinfect WW are chlorination, ozonation, and UV irradiation [73]. Although chlorine-based agents and ozone lead to complete viral inactivation, their application may be expensive and can lead to the formation of toxic subproducts. On the other hand, UV irradiation is less effective than chlorination and ozonation, and the outcome is highly influenced by the structure of the viral particles [73]. Recent studies showed that coronaviruses exhibit higher resistance to this treatment when compared to other viruses [73]. Furthermore, for the efficient inactivation of the SARS coronavirus, high doses of light (above 1176 mJ/cm^2^) are required [73]. In comparison, the PDI approach also showed a great viral inactivation efficiency that was similar to that of the chlorination and ozonation techniques, promoting the complete inactivation of bacteriophage φ6 after just 10 min of treatment, without leading to the formation of toxic subproducts or promoting the development of resistant strains.

Contrarily to the conventional tertiary treatments applied in the disinfection of WW, the PDI approach is efficient in microbial inactivation without the production of known toxic subproducts, without inducing genetic damage to microorganisms, and, consequently, without contributing to the development of resistant strains. In order to develop a safe WW treatment protocol, it is crucial to understand if PDI-treated effluents would affect cultivable native marine aquatic microorganisms where they are discharged. The results of this study showed that only in the case of the less diluted samples (1:2) that were subjected to white-light irradiation (S, light), the viability of the native microorganisms was negatively affected. These results are expected, since the PSs still present in these less diluted light samples are able to produce ROSs after being activated by light. However, at higher dilutions, this effect was no longer significant, as the concentration of PSs present in the samples was much lower. As the WW is greatly diluted when it is discharged into the marine environment, the potential impact on the aquatic organisms should not be significant. In a study conducted by Ramos and Neves [74], the authors monitored the discharge of wastewater occurring through an underwater sewage outlet (in the central coastal region of Portugal). With data collected with the help of an autonomous underwater vehicle, the authors presented predictive mathematical models based on the theory of jets (effluent behavior close to the source of flow) and plumes (resulting from the behavior of the effluent far from the source of flow) and were able to map the dispersion of the plume and its dilution as a function of physicochemical parameters, such as the temperature of the effluent and the receiving waters, as well as salinity differences. Additionally, ocean currents play an important role in the dispersion/dilution of effluents in receiving waters. In the given submarine wastewater pipe, the average discharge flow rate is about 0.8 m^3^ s^−1^ and occurs at a depth of about 15 m. The dilution estimates are consistently greater than 30 (in the initial mixing zone, between 15 and 11 m depth), with plume dilution being estimated at more than 300 up to a depth of 8 m.

The main limitation of the applicability of this approach to the disinfection of WW in WWTPs relies on the desirable retention of PS molecules after treatment. However, to surpass this limitation, PS molecules can be immobilized in solid supports (e.g., nanomagnet particles, silica, cellulose, chitosan) that allow their recovery from WW after treatment [41,75,76,77]. In addition, the possible reuse of immobilized PSs in further treatments makes PDI not only environmentally friendly, but also cost effective.

Nevertheless, more studies are needed in order to evaluate the probable impact that PDI treatment could have if applied in a real context in a WWTP.

## 5. Conclusions

In general, it can be concluded that (i) the phage φ6 (used as a SARS-CoV-2 surrogate) remains viable in the environment for a considerable amount of time under conditions similar to those of environments in which WW is released after treatment and of the WWTPs where WW is treated; (ii) the PDI process is effective in inactivating phage φ6 in WW; (iii) at higher dilution rates in seawater, the treated effluent does not cause toxicity to cultivable native marine aquatic microorganisms.

It is important to note that the results were obtained for phage φ6, and although phage φ6 is considered a suitable model of SARS-CoV-2, further studies using SARS-CoV-2 are needed. Nevertheless, this study opens the opportunity to consider PDI as a potential and effective WW treatment for controlling emergent viruses and diminishing the risks of outbreak through spread in the environment.

## Data Availability

Not applicable.
